# Impact of the Choice of Native T_1_ in Pixelwise Myocardial Blood Flow Quantification

**DOI:** 10.1002/jmri.27375

**Published:** 2020-10-08

**Authors:** Corina Kräuter, Ursula Reiter, Clemens Reiter, Volha Nizhnikava, Albrecht Schmidt, Rudolf Stollberger, Michael Fuchsjäger, Gert Reiter

**Affiliations:** ^1^ Division of General Radiology, Department of Radiology Medical University of Graz Graz Austria; ^2^ Institute of Medical Engineering Graz University of Technology Graz Austria; ^3^ Division of Cardiology, Department of Internal Medicine Medical University of Graz Graz Austria; ^4^ Research and Development Siemens Healthcare Diagnostics GmbH Graz Austria

**Keywords:** myocardial blood flow, cardiovascular magnetic resonance, nonlinearity correction, T1 mapping, Fermi modeling, dynamic contrast enhancement

## Abstract

**Background:**

Quantification of myocardial blood flow (MBF) from dynamic contrast‐enhanced (DCE) MRI can be performed using a signal intensity model that incorporates T_1_ values of blood and myocardium.

**Purpose:**

To assess the impact of T_1_ values on pixelwise MBF quantification, specifically to evaluate the influence of 1) study population‐averaged vs. subject‐specific, 2) diastolic vs. systolic, and 3) regional vs. global myocardial T_1_ values.

**Study Type:**

Prospective.

**Subjects:**

Fifteen patients with chronic coronary heart disease.

**Field Strength/Sequence:**

3T; modified Look–Locker inversion recovery for T_1_ mapping and saturation recovery gradient echo for DCE imaging, both acquired in a mid‐ventricular short‐axis slice in systole and diastole.

**Assessment:**

MBF was estimated using Fermi modeling and signal intensity nonlinearity correction with different T_1_ values: study population‐averaged blood and myocardial, subject‐specific systolic and diastolic, and segmental T_1_ values. Myocardial segments with perfusion deficits were identified visually from DCE series.

**Statistical Tests:**

The relationships between MBF parameters derived by different methods were analyzed by Bland–Altman analysis; corresponding mean values were compared by *t*‐test.

**Results:**

Using subject‐specific diastolic T_1_ values, global diastolic MBF was 0.61 ± 0.13 mL/(min·g). It did not differ from global MBF derived from the study population‐averaged T_1_ (*P* = 0.88), but the standard deviation of differences was large (0.07 mL/(min·g), 11% of mean MBF). Global diastolic and systolic MBF did not differ (*P* = 0.12), whereas global diastolic MBF using systolic (0.62 ± 0.13 mL/(min·g)) and diastolic T_1_ values differed (*P* < 0.05). If regional instead of global T_1_ values were used, segmental MBF was lower in segments with perfusion deficits (bias = −0.03 mL/(min·g), −7% of mean MBF, *P* < 0.05) but higher in segments without perfusion deficits (bias = 0.01 mL/(min·g), 1% of mean MBF, *P* < 0.05).

**Data Conclusion:**

Whereas cardiac phase‐specific T_1_ values have a minor impact on MBF estimates, subject‐specific and myocardial segment‐specific T_1_ values substantially affect MBF quantification.

**Level of Evidence:**

3

**Technical Efficacy Stage:**

3

DYNAMIC CONTRAST‐ENHANCED (DCE) cardiac magnetic resonance imaging (MRI) represents a recognized technique for the assessment of myocardial perfusion and ischemia.^1^, ^2^ Whereas a series of longitudinal relaxation time (T_1_)‐weighted images acquired during the first passage of a bolus of contrast agent (CA) are typically inspected visually for regional myocardial perfusion deficits, corresponding temporal signal intensity (SI) changes might be employed not only to derive semiquantitative perfusion‐related parameters but also to estimate quantitative myocardial blood flow (MBF) in units of mL/(min·g).^3^, ^4^

MBF relates temporal CA concentration changes in the left ventricular (LV) blood pool, represented by the arterial input function (AIF), with temporal CA concentration changes in myocardial tissue.^3^, ^4^ Consequently, MBF estimation requires the conversion of measured SI in blood and myocardial tissue into corresponding CA concentrations, which is complicated by their typically nonlinear relationships.^4^, ^5^ Various conversion methods have been introduced, including minimization of deviation from linearity directly during image acquisition, as performed by low‐dosage, dual‐bolus, and dual‐sequence approaches, the use of retrospectively empirical calibration curves, or the application of SI models in combination with additional calibration images.^6^, ^7^, ^8^, ^9^, ^10^, ^11^, ^12^, ^13^, ^14^

The usage of an SI model that incorporates native T_1_ values of blood and myocardium for SI‐to‐CA‐concentration conversion is tempting because of its universality and the integration of T_1_ mapping into clinical cardiovascular MRI.^15^ While MBF estimation has been extensively studied employing native T_1_ normal values, the first attempts were made to render the method more subject‐specific by using native blood and myocardial T_1_ values measured from native T_1_ maps.^14^, ^16^, ^17^, ^18^, ^19^, ^20^, ^21^ The feasibility of pixelwise MBF quantification employing an SI model incorporating native T_1_ values of blood and myocardium remains, however, to be examined. Studies show that native myocardial T_1_ values differ between systole and diastole as well as between myocardial segments, the latter especially if infarcted regions are present.^22^, ^23^, ^24^ We therefore hypothesize that not only global, but also regional native myocardial T_1_ values, as well as the cardiac phase of native T_1_ maps, affect SI model‐based MBF estimates.

Thus, the aims of this study were to assess the impact of native T_1_ values on pixelwise MBF quantification by evaluating 1) the necessity for using subject‐specific native T_1_ values, 2) the influence of the cardiac phase, and 3) the need for using regional instead of global native myocardial T_1_ values.

## Materials and Methods

### *Study Population*

This prospective study (ClinicalTrials.gov identifier, NCT03253835) was approved by the local Ethical Review Board, and all subjects gave written informed consent. Between February 2016 and April 2017, 20 adult patients with known chronic coronary heart disease (CHD) and without contraindications to contrast‐enhanced MR underwent comprehensive cardiac MRI including myocardial DCE imaging and native and postcontrast T_1_ mapping. To allow clear assignment of DCE series and T_1_ maps to a cardiac phase, five subjects were excluded from analysis because of arrhythmia or erroneous electrocardiographical (ECG) triggering during DCE imaging or T_1_ mapping. Thus, the analyzed patient population consisted of 15 patients (13 male, 2 female).

### *MR Image Acquisition*

ECG‐gated cardiac MR was performed with a 3T clinical MR scanner (Magnetom Skyra, Siemens Healthcare, Erlangen, Germany) using an 18‐channel body coil and 12 elements of a 32‐channel spine coil with the patient in the supine position. T_1_ maps and DCE series were acquired under a resting condition and inspiratory breath‐holding in the same mid‐ventricular short‐axis slice.

An ECG‐gated modified Look–Locker inversion recovery (MOLLI) sequence with single‐shot balanced steady‐state free precession (bSSFP) readout, motion correction, and automatic T_1_ map generation was used to derive native T_1_ maps at end‐systole and diastasis. The MOLLI scheme was 5(5)3, meaning the acquisition of five images after the first inversion pulse and after a waiting period of five heartbeats the acquisition of three further images after a second inversion pulse.^25^, ^26^ Protocol parameters of the bSSFP readout were repetition time (TR) = 2.7 msec; echo time (TE) = 1.1 msec; flip angle = 35°; bandwidth = 1085 Hz/pixel; generalized autocalibrating partially parallel acquisition (GRAPPA) factor = 2; partial Fourier reconstruction = 7/8; field of view (FOV) = 307 × 360 mm^2^; and voxel size = 2.1 × 1.4 × 8.0 mm^3^.

DCE imaging was performed with the CA gadobutrol (Gadovist, Bayer Schering Pharma, Berlin, Germany) at a dose of 0.05 mmol/kg body weight. The CA was administered into the right antecubital vein by means of a power injector (Medrad, Volkach, Germany) at a rate of 4 mL/s, followed by a saline flush of 30 mL at the same rate. Starting with CA administration, its passage was imaged for 70 heartbeats employing an ECG‐gated single‐shot saturation recovery fast low‐angle shot (SR FLASH) sequence; the breath‐hold command given during acquisition targeted a breath‐hold period from the arrival of the bolus in the LV until its second pass. The imaging time per frame of the SR FLASH sequence was 158.8 msec, which allowed imaging of the same mid‐ventricular short‐axis slice four to six times within each cardiac interval. Further protocol parameters were TR = 2.2 msec; TE = 1.1 msec; time between composite saturation pulse and central *k*‐space line of image readout (TI) = 90 msec; flip angle = 12°; bandwidth = 930 Hz/pixel; GRAPPA factor = 2; FOV = 330 × 360 mm^2^; voxel size = 2.7 × 1.9 × .0 mm^3^; and matrix = 124 × 192. Images of the DCE scan within the first two heartbeats were acquired without applying magnetization preparation at a low flip angle of 5°. The resulting (precontrast) proton density‐weighted images were used to estimate the coil sensitivity of the receiver coils. This estimation, together with a surface coil correction as well as a nonrigid motion correction of every frame, was performed automatically during image reconstruction by the scanner software.^27^

Immediately after the DCE scan, a second bolus of CA was administered at a dose of 0.10 mmol/kg body weight. Approximately 15 minutes after CA administration, myocardial late gadolinium enhancement (LGE) was imaged by T_1_ mapping at diastasis employing the MOLLI sequence described above but with MOLLI scheme 4(1)3(1)2 (acquisition of 4, 3, and 2 images after the first, second, and third inversion pulse, respectively, with waiting periods of one heartbeat in between).^26^

For detailed patient characterization, cardiac MR also included LV function and LGE imaging in inspiratory breath‐holding. For details, see the Supporting Information.

### *Visual Analysis of Regional Myocardial Perfusion Deficits*

**FIGURE 1:**
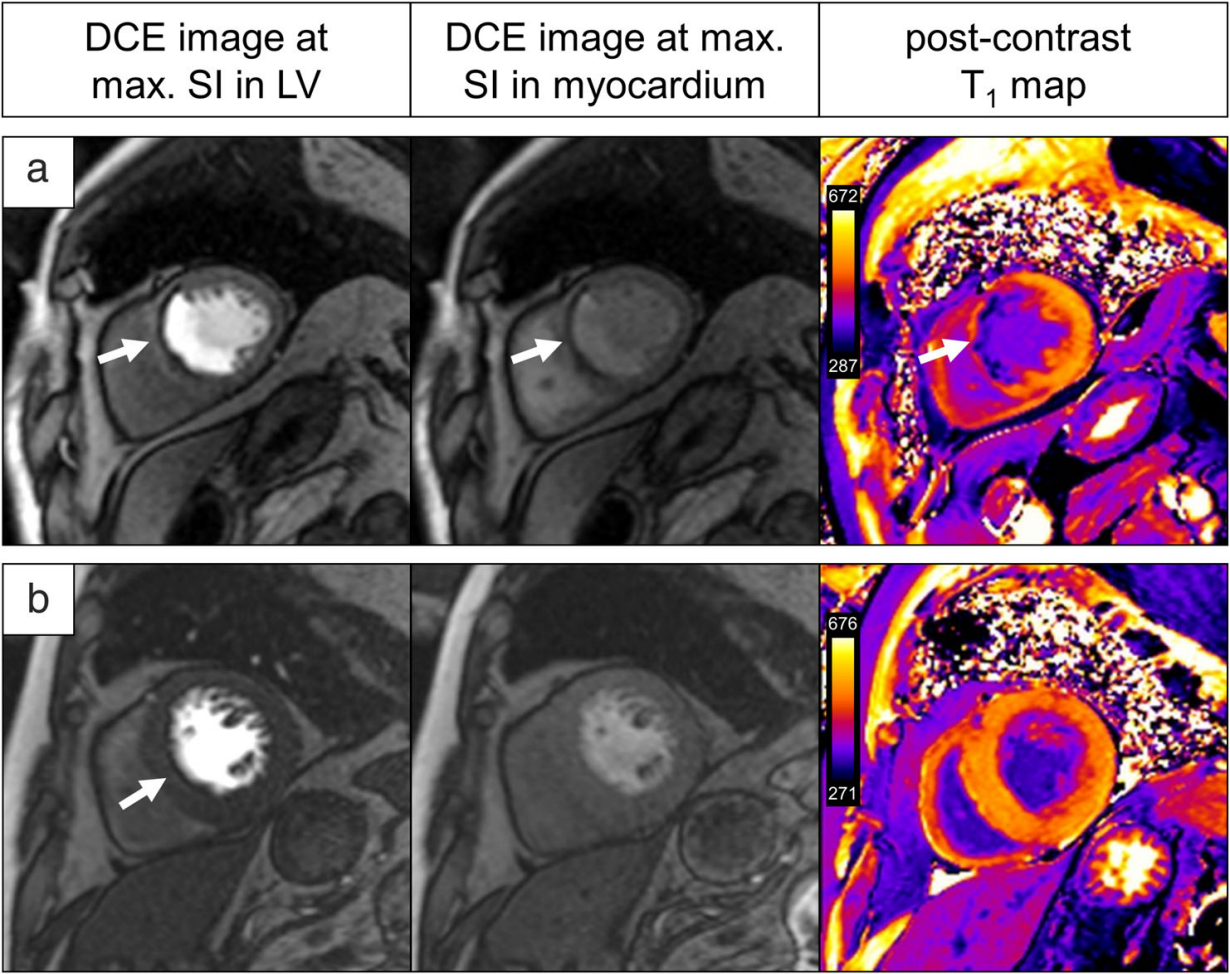
Example of a perfusion deficit (**a**) and a dark rim artifact (**b**). In both cases, the DCE image during maximum SI in the LV (left panel) shows reduced SI increase in the myocardium (arrow). During maximum SI in the myocardium or equivalently some seconds later (center panel), only the perfusion deficit persists. Only the perfusion deficit demonstrates regional consistent late gadolinium enhancement in the postcontrast T_1_ map (right panel). DCE = dynamic contrast‐enhanced; SI = signal intensity; LV = left ventricle.

The mid‐ventricular DCE series acquired in diastole were analyzed visually for regional myocardial perfusion deficits, which were interpreted according to the American Heart Association (AHA) model by three readers (C.R., V.N., and U.R. with 4, 5, and 20 years of experience, respectively). The discrimination between perfusion deficits and possible dark rim artifacts was based on localization and duration of reduced SI increase during the first passage of CA as well as the presence of LGE visualized in postcontrast T_1_ maps (Fig. 1).^3^ In particular, an AHA segment was counted as a perfusion deficit segment if the majority of readers identified reduced SI increase in at least 50% along the subendocardial border of the segment.

### *Determination of Regional Native T_1_ Values*

Systolic and diastolic global native myocardial and blood T_1_ values were determined by manual segmentation of the endocardial and epicardial borders, as well as a region of interest (ROI) placed in the LV blood pool in the corresponding native T_1_ maps by a reader with 4 years of experience (C.K.) using dedicated cardiac image analysis software (cvi^42^, Circle Cardiovascular Imaging, Calgary, Canada). An offset of 25% from the drawn endo‐ and epicardial contours was chosen to ensure robust T_1_ mean value estimates.^28^ Moreover, diastolic T_1_ maps were evaluated according to the six AHA segments of the measured mid‐ventricular slice, halves of these AHA segments, thirds of these AHA segments, and quarters of these AHA segments to derive the corresponding 12‐, 18‐, and 24‐sectional mean native T_1_ values.

### *Pixelwise and Regional MBF Determination*

**FIGURE 2:**
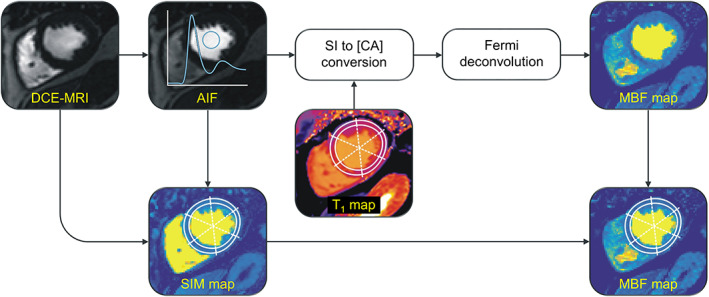
Overview of the image processing steps for pixelwise and regional MBF quantification. The motion and coil sensitivity corrected perfusion MR data is first used to obtain the AIF from an ROI placed on one image with good contrast between LV blood pool and myocardium. Features of the extracted AIF are employed as temporal landmarks when generating a signal intensity maximum (SIM) map, which is used as a base image for the segmentation of the myocardium. The AIF and every single pixel of the perfusion series are converted from SI to CA concentration using SI model‐based nonlinearity correction and incorporating native T_1_ values measured from a precontrast T_1_ map. Pixelwise MBF is determined employing Fermi function constrained deconvolution. DCE‐MRI = dynamic contrast‐enhanced magnetic resonance imaging; AIF = arterial input function; SI = signal intensity; CA = contrast agent; [CA] = contrast agent concentration; SIM = signal intensity maximum; MBF = myocardial blood flow; ROI = region of interest.

DCE series in end‐systole (systolic DCE series) and diastasis (diastolic DCE series) together with an estimate of native myocardial and blood T_1_ values were converted to pixelwise MBF maps and regional MBF estimates using in‐house software implemented in MATLAB (MathWorks, Natick, MA). Figure 2 shows an overview of the image processing steps.

In a first step, the AIF was obtained from the DCE series as the mean signal from a central ROI in the LV blood pool, which was manually drawn onto one image of the perfusion series with suitable contrast between LV blood pool and myocardium, excluding papillary muscles. Then temporal SI changes of the AIF and every single pixel of the DCE series were converted to CA concentration changes using an SI model for the employed SR FLASH sequence^6^, ^29^:

(1) $$\mathrm{SI}=c∙\left[\left(1-e^{-\mathrm{TD}/T_{1}}\right)∙a^{n-1}+\left(1-e^{-\mathrm{TR}/T_{1}}\right)∙\frac{1-a^{n-1}}{1-a}\right]\;\text{with}\;a=\cos\left(\alpha \right)∙e^{-\mathrm{TR}/T_{1}}$$

where *α* denotes the flip angle, *TR* the repetition time per phase encoding, *n* the number of phase encoding steps between acquisition start and *k*‐space center (set to 31), and *TD* the time delay between the composite saturation pulse and the start of FLASH readout (set to 21.2 msec). *c* is a scaling factor proportional to the equilibrium magnetization and is assumed to be constant throughout the DCE series.^11^ For every pixel, *c* was estimated employing the native T_1_ value of blood or myocardium (for choices of native T_1_ values, see next section) as well as the baseline signal determined as the temporal median of the signal from start to CA arrival in the LV blood pool. Equation 1 was then used to determine the T_1_ values for each timepoint of the AIF and each pixel of the DCE series. CA concentrations corresponding to the calculated T_1_ values were determined using the relationship:

(2) $$\frac{1}{T_{1}}=\frac{1}{T_{1,0}}+r_{1}∙\left[\mathrm{CA}\right]$$

where *T*_1,0_ denotes the native T_1_ estimate, *r*_1_ the T_1_ relaxivity constant of the contrast agent, and [CA] the contrast agent concentration. *r*_1_ was set at 5.0 L·mmol^−1^·s^−1^ and assumed to remain unchanged when the CA passed from blood into tissue.^4^, ^30^

Pixelwise MBF was quantified using Fermi function model constrained deconvolution, employing the AIF of the respective cardiac phase (eg, for DCE series in systole, the systolic AIF, which was converted to CA concentration with the systolic native T_1_ value).^6^ In doing so, the DCE series was restricted to the first passage of CA, with the end of the first passage identified as the valley point after the maximum of the AIF. The time shift between AIF and myocardial signal was identified as the one yielding the minimal fitting error between the deconvolution‐determined and measured myocardial signal.^31^ Global and segmental MBF values were calculated as the mean MBF of all pixels of the corresponding myocardial region; without further specification, MBF refers to global MBF.

### *Experiments on the Impact of Native T_1_ Values on MBF*

At the SI‐to‐CA‐concentration conversion processing step, several experiments were performed to evaluate the influence of native T_1_ values on pixelwise MBF estimates. MBF maps were determined:

1. without nonlinearity correction (by only subtracting the baseline SI),
2. using ranges of native blood and myocardial T_1_ normal values at 3T as well as the study population‐averaged native blood and global myocardial T_1_ values,^32^, ^33^, ^34^, ^35^, ^36^
3. employing individual patients' native blood and global myocardial T_1_ values,
4. for the perfusion series in the systolic and diastolic phase, one time using native T_1_ values of the respective cardiac phase and one time using the native T_1_ values of the other cardiac phase,
5. using successively smaller native myocardial T_1_ regions, ranging from six AHA segments of the mid‐ventricular slice up to 24 sections.

Apart from the cardiac phase comparisons (experiment 4), all evaluations were performed employing diastolic DCE series. In experiments 3 and 5, diastolic native T_1_ values were used. Additionally, the impact of the cardiac phase in which the AIF was measured was investigated by applying the systolic AIF to diastolic DCE series in experiments 1–4.

### *Statistical Analysis*

Statistical analysis was performed with MedCalc (MedCalc Software, Ostend, Belgium), considering *P* < 0.05 as significant. Mean values are specified together with standard deviations.

Equality of variances of distributions of perfusion and T_1_ parameters was tested by the variance ratio test, their normality by the Kolmogorov–Smirnoff test. Study population mean values of perfusion and T_1_ parameters were compared by paired *t*‐test or by Wilcoxon test, in case of nonnormality of the differences. Differences between perfusion and T_1_ parameters in AHA segments with and without perfusion deficits were analyzed by means of unpaired *t*‐test, Welch test, in case of unequal variances, or Mann–Whitney test, in case of nonnormality. Relationships between MBF and T_1_ parameters were analyzed by the Pearson correlation coefficient (*r*) and its significance level. Relationships between global, segmental, and pixelwise MBF parameters derived by different methods were studied again by Pearson correlation analysis as well as linear regression and Bland–Altman analysis.

## Results

### *Study Population*

Demographic as well as LV function parameters of the study population are summarized in Supporting Information Table S1.

Whereas systolic and diastolic native blood T_1_ values (1897 ± 138 msec vs. 1891 ± 136 msec) did not differ significantly (*P* = 0.07), systolic and diastolic global native myocardial T_1_ values (1249 ± 65 msec vs. 1259 ± 80 msec) differed (*P* < 0.05). However, systolic and diastolic native T_1_ values correlated strongly (*r* = 1.00 for blood and *r* = 0.99 for myocardium, *P* < 0.05 in both cases).

Myocardial perfusion deficits were identified in seven patients and 13 AHA segments; except for one AHA segment, this was agreed upon by all three observers. All perfusion deficits were surrounded by LGE (for further details, see Supporting Information). Mean diastolic native T_1_ values in myocardial segments with perfusion deficits were significantly higher than in segments without perfusion deficits (1425 ± 170 msec vs. 1235 ± 66 msec, *P* < 0.05).

### *MBF Quantification Without Subject‐Specific Native T_1_ Values*

MBF determined without nonlinearity correction was significantly higher than MBF determined using subject‐specific global native T_1_ values (MBF SI = 0.71 ± 0.11 mL/(min·g) vs. MBF subject‐specific T_1_ = 0.61 ± 0.13 mL/(min·g), *P* < 0.05) and they correlated strongly (*r* = 0.85, *P* < 0.05).

**FIGURE 3:**
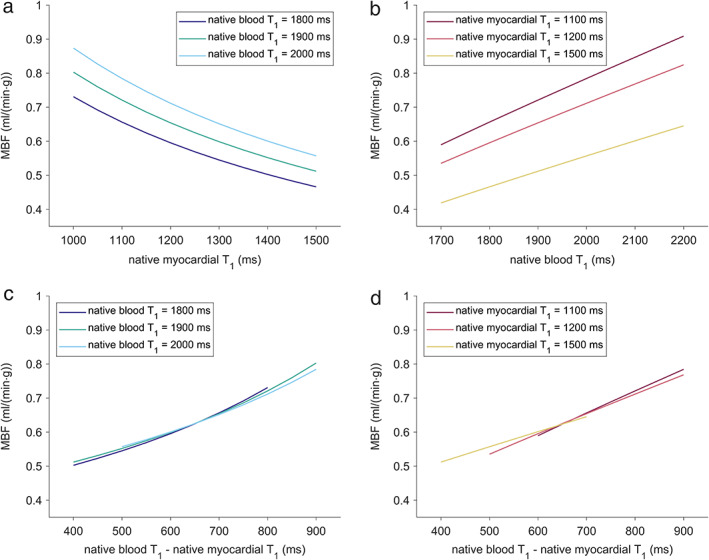
MBF determined using normal ranges of native blood and myocardial T_1_ values at 3T. The mean of MBF of all patients at varying myocardial T_1_ values while keeping the blood T_1_ value fixed (**a**) and at varying blood T_1_ values while keeping the myocardial T_1_ value fixed (**b**). The mean of MBF of all patients at varying differences between blood and myocardial T_1_ values while keeping the blood T_1_ value fixed (**c**) and while keeping the myocardial T_1_ value fixed (**d**). MBF = myocardial blood flow.

**FIGURE 4:**
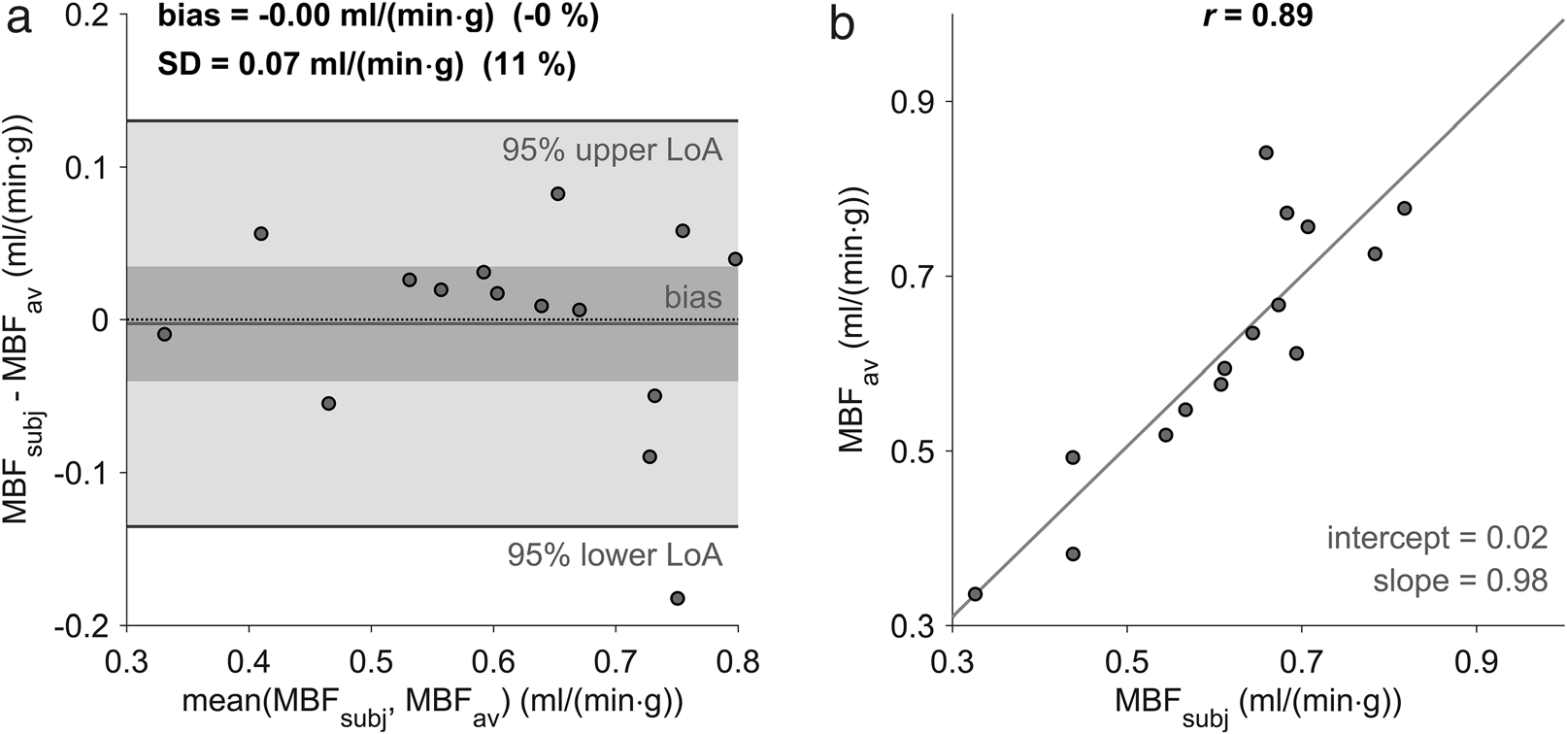
Bland–Altman (**a**) and linear regression (**b**) plots for comparison of MBF estimates determined with subject‐specific native T_1_ values and the study population‐averaged native T_1_ values. The dark gray bar indicates the 95% confidence limits of the bias. MBF = myocardial blood flow; MBF_subj_ = MBF with subject‐specific native T_1_ values; MBF_av_ = MBF with study population‐averaged native T_1_ values; SD = standard deviation of differences; LoA = limits of agreement; *r* = Pearson correlation coefficient.

Using the range of native myocardial and blood T_1_ normal values for MBF estimation yielded lower mean MBF for higher native myocardial T_1_ values and higher mean MBF for higher native blood T_1_ values (Fig. 3a,b). Figure 3c,d demonstrate that mean MBF becomes higher predominantly with the difference of native blood and myocardial T_1_ values, irrespective of absolute blood or myocardial T_1_ values. The mean MBF determined with the study population‐averaged diastolic native T_1_ values resulted in 0.62 ± 0.15 mL/(min·g). Whereas this mean value did not differ from the mean MBF determined with subject‐specific native T_1_ values (*P* = 0.88) and MBF estimates correlated strongly (*r* = 0.89, *P* < 0.05), MBF values of single patients deviated substantially from the mean difference (Fig. 4).

Of note, whereas mean MBF estimates were lower for higher native myocardial T_1_ normal values and lower native blood T_1_ normal values, the subject‐specific MBF values did not correlate significantly either with the subject‐specific native myocardial (*r* = 0.26, *P* = 0.35) or blood T_1_ values (*r* = 0.12, *P* = 0.64) or their difference (*r* = 0.04, *P* = 0.88).

### *MBF Quantification With Systolic and Diastolic Native T_1_ Values*

**FIGURE 5:**
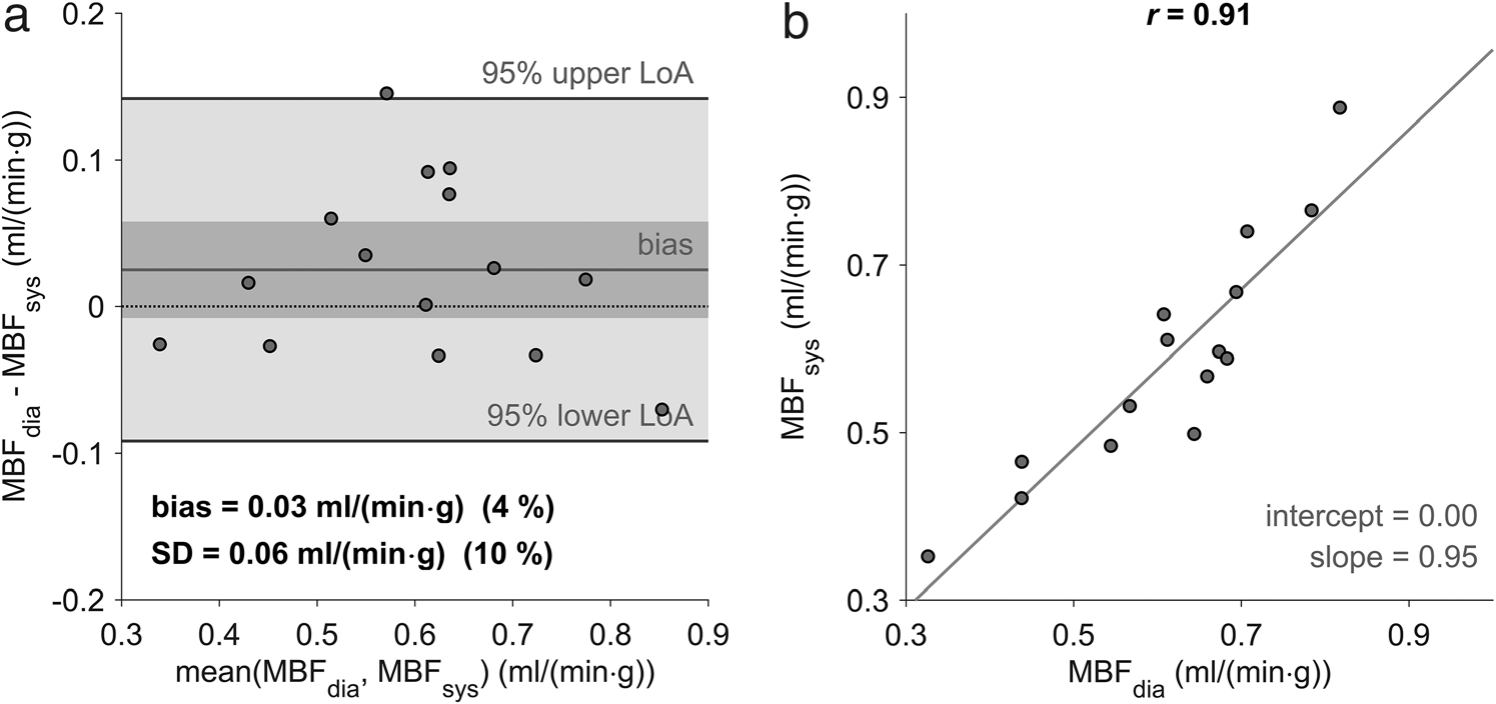
Bland–Altman (**a**) and linear regression (**b**) plots for comparison of systolic and diastolic MBF estimates determined with systolic and diastolic native T_1_ values, respectively. The dark gray bar indicates the 95% confidence limits of the bias. MBF = myocardial blood flow; MBF_dia_ = diastolic MBF with diastolic native T_1_ values; MBF_sys_ = systolic MBF with systolic native T_1_ values; SD = standard deviation of differences; LoA = limits of agreement; *r* = Pearson correlation coefficient.

**FIGURE 6:**
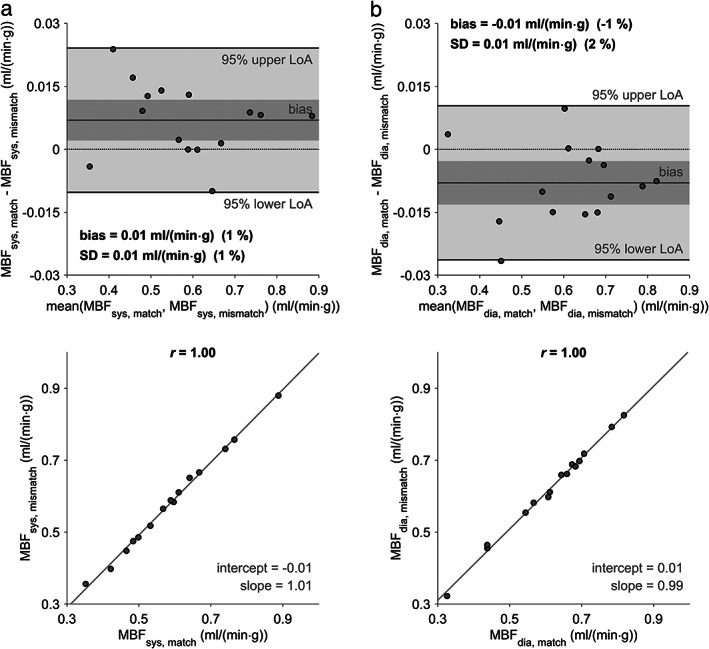
Bland–Altman and linear regression plots for comparison of systolic (**a**) and diastolic (**b**) MBF estimates determined with native T_1_ values of the respective cardiac phase and native T_1_ values of the other cardiac phase. The dark gray bar indicates the 95% confidence limits of the bias. MBF = myocardial blood flow; MBF_sys, match_ = systolic MBF with systolic native T_1_ values; MBF_sys, mismatch_ = systolic MBF with diastolic native T_1_ values; MBF_dia, match_ = diastolic MBF with diastolic native T_1_ values; MBF_dia, mismatch_ = diastolic MBF with systolic native T_1_ values; SD = standard deviation of differences; LoA = limits of agreement; *r* = Pearson correlation coefficient.

Systolic MBF determined using systolic native myocardial and blood T_1_ values did not differ significantly from diastolic MBF determined with diastolic native myocardial and blood T_1_ values (systolic MBF with systolic T_1_ = 0.59 ± 0.14 mL/(min·g) vs. diastolic MBF with diastolic T_1_ = 0.61 ± 0.13 mL/(min·g), *P* = 0.12). Moreover, a strong correlation between systolic and diastolic MBF estimates was observed (Fig. 5). However, MBF estimates calculated from native T_1_ values at different cardiac phases differed significantly (systolic MBF with systolic T_1_ = 0.59 ± 0.14 mL/(min·g) vs. systolic MBF with diastolic T_1_ = 0.58 ± 0.14 mL/(min·g), *P* < 0.05; diastolic MBF with systolic T_1_ = 0.62 ± 0.13 mL/(min·g) vs. diastolic MBF with diastolic T_1_ = 0.61 ± 0.13 mL/(min·g), *P* < 0.05). Bland–Altman and regression plots of the comparisons are given in Fig. 6.

The maximum values of systolic and diastolic AIFs were not significantly different, either for SI curves (systolic AIF_max_ = 663 ± 194 vs. diastolic AIF_max_ = 657 ± 187, *P* = 0.53) or for CA concentration signals (systolic AIF_max_ = 3.72 ± 1.14 mmol/L vs. diastolic AIF_max_ = 3.67 ± 1.13 mmol/L, *P* = 0.64). Correspondingly, using the systolic AIF instead of the AIF of the respective cardiac phase did not change the results of the global MBF comparisons above (MBF SI vs. MBF subject‐specific T_1_, MBF study population‐averaged T_1_ vs. MBF subject‐specific T_1_, systolic MBF vs. diastolic MBF, systolic MBF with systolic T_1_ vs. systolic MBF with diastolic T_1_, diastolic MBF with diastolic T_1_ vs. diastolic MBF with systolic T_1_).

### *MBF Quantification Using Regional Native Myocardial T_1_ Values*

**FIGURE 7:**
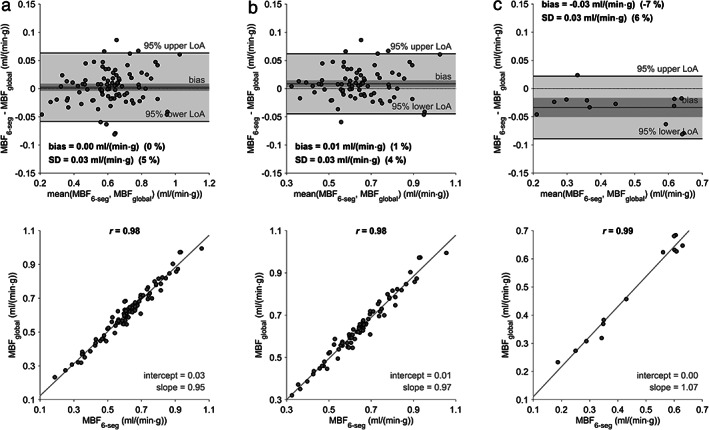
Bland–Altman and linear regression plots comparing 6‐segmental mean MBF values determined with global and 6‐segmental native myocardial T_1_ values. MBF of all segments of all patients (**a**), all segments without perfusion deficits (**b**), and all segments exhibiting perfusion deficits (**c**). The dark gray bar indicates the 95% confidence limits of the bias. MBF = myocardial blood flow; MBF_global_ = MBF with global native T_1_ values; MBF_6‐seg_ = MBF with 6‐segmental native T_1_ values; SD = standard deviation of differences; LoA = limits of agreement; *r* = Pearson correlation coefficient.

Global MBF estimates determined with global native myocardial T_1_ values did not differ significantly from global MBF estimates determined with AHA‐segmental (6‐segmental) native myocardial T_1_ values (MBF global T_1_ = 0.61 ± 0.13 mL/(min·g) vs. MBF 6‐segmental T_1_ = 0.61 ± 0.13 mL/(min·g), *P* = 0.11). The comparison of all 6‐segmental MBF values calculated with global and 6‐segmental native myocardial T_1_ values also did not yield a significant difference (*P* = 0.44; Fig. 7a). However, if the segments without and with perfusion deficits were considered individually, a significant difference between 6‐segmental MBF estimates determined with global and 6‐segmental native myocardial T_1_ values was observed: Segments without perfusion deficits showed higher MBF when 6‐segmental instead of global native myocardial T_1_ values were used (*P* < 0.05; Fig. 7b), while segments with perfusion deficits exhibited lower MBF (*P* < 0.05; Fig. 7c).

**FIGURE 8:**
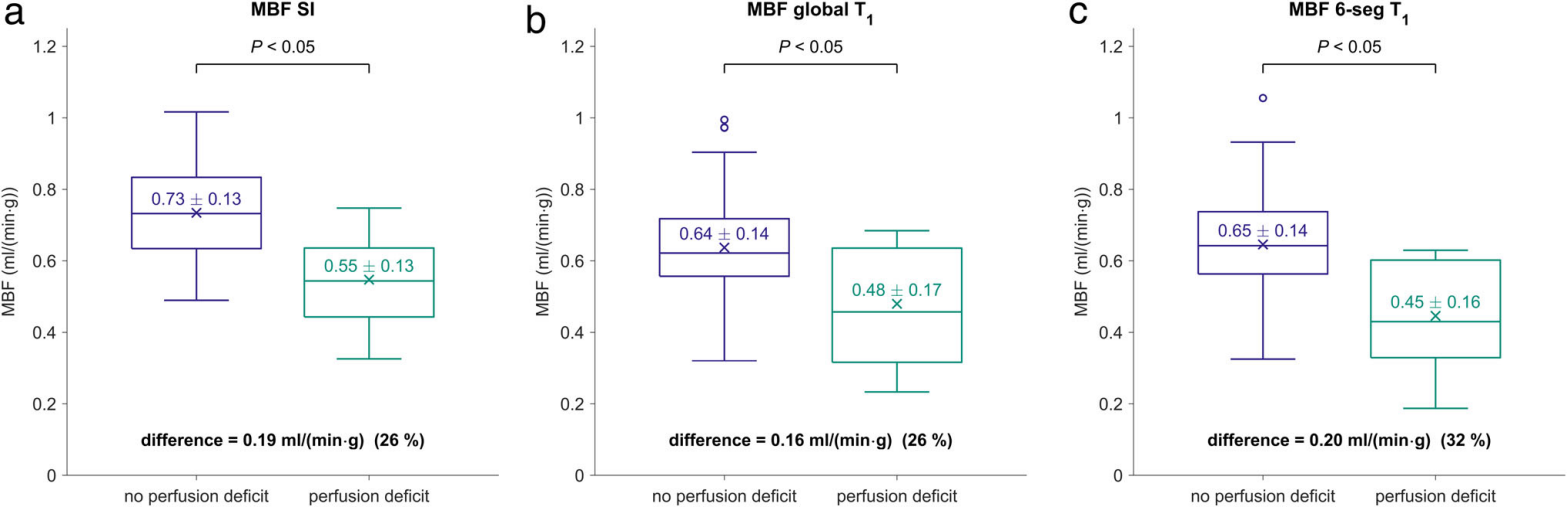
Boxplots of MBF in segments with and without perfusion deficits, calculated without nonlinearity correction (**a**), with global native T_1_ values (**b**), and with 6‐segmental native T_1_ values (**c**). Mean MBF is indicated by × and stated with the standard deviation, *P* refers to the comparison of the means. MBF = myocardial blood flow; MBF SI = MBF without nonlinearity correction; MBF global T_1_ = MBF with global native T_1_ values; MBF 6‐seg T_1_ = MBF with 6‐segmental native T_1_ values.

Figure 8 demonstrates boxplots of MBF estimates in segments with and without perfusion deficits, calculated without nonlinearity correction, with global native T_1_ values and with 6‐segmental native T_1_ values. MBF in segments with perfusion deficits was significantly lower than MBF in segments without perfusion deficits in all cases. However, the mean difference of MBF between segments with and without perfusion deficits was smaller in the case of MBF calculated with global native T_1_ and larger in the case of 6‐segmental native T_1_ when comparing to MBF determined without nonlinearity correction. Comparisons of pixelwise MBF values determined with a successively larger number of native myocardial T_1_ sections revealed that the largest impact of T_1_‐sectioning occurs from global to 6‐segmental, considering the pixelwise MBF correlations and standard deviations of pixelwise differences (Table 1).

**TABLE 1:** Comparisons of Pixelwise MBF Values of All Patients Determined With a Successively Larger Number of Native Myocardial T_1_ Sections

Comparisons of pixelwise MBF	*r*	Difference of MBF (mL/(min·g))	*P* value
Global T_1_ vs. 6‐segmental T_1_	0.99	0.0015 ± 0.0306 (0.25 ± 5.05%)	<0.05
6‐segmental T_1_ vs. 12‐sectional T_1_	1.00	0.0012 ± 0.0159 (0.19 ± 2.61%)	<0.05
12‐sectional T_1_ vs. 18‐sectional T_1_	1.00	0.0004 ± 0.0141 (0.07 ± 2.32%)	0.33
18‐sectional T_1_ vs. 24‐sectional T_1_	1.00	0.0002 ± 0.0114 (0.04 ± 1.87%)	0.10

*r* indicates the Pearson correlation coefficient; *P* value refers to Wilcoxon test.

## Discussion

The main findings of this study are 1) pixelwise MBF quantification using native T_1_ mapping for SI model‐based nonlinearity correction is feasible; 2) using native T_1_ normal values for blood and myocardium may lead to large variations in MBF estimates; 3) the cardiac phase in which native T_1_ values are acquired significantly affects MBF estimates, even though the resulting MBF bias is small; and 4) MBF should be determined using regional instead of global native T_1_ values.

### *Pixelwise MBF Quantification*

While previous studies on pixelwise MBF estimation in humans either used SI nonlinearity correction at image acquisition or employed native T_1_ normal values for the SI model, the present study determined pixelwise MBF employing a clinical DCE protocol in combination with routinely acquired subject‐specific native T_1_ maps for SI model‐based nonlinearity correction.^9^, ^19^, ^37^ Both the average global MBF value of our study population and the mean MBF value in segments without perfusion deficits were within the range of published rest perfusion estimates (0.51 mL/(min·g) to 1.03 L(min·g)), which shows that the employed SI nonlinearity correction method yields reasonable MBF estimates.^9^, ^37^ The comparably low MBF values of the present study can be explained by chronic CHD patients being very likely to exhibit (globally) reduced myocardial perfusion even at rest.

### *MBF Quantification Without Subject‐Specific Native T_1_ Values*

As observed previously, MBF estimates determined without nonlinearity correction were significantly higher than those determined using SI model‐based nonlinearity correction with subject‐specific native T_1_ values.^19^ Interestingly, employing native T_1_ normal values in nonlinearity correction (experiment 2) might result in an even higher MBF bias.

Experiment 2 also showed that variations in native myocardial and blood T_1_ values have a similarly strong effect on MBF estimates. It is therefore necessary to measure both native myocardial and blood T_1_ values subject‐wise instead of using fixed native T_1_ reference values. This is further supported by the large limits of agreement in the Bland–Altman plot comparing MBF estimates determined with the study population‐averaged native T_1_ values and subject‐specific native T_1_ values.

### *MBF Quantification With Systolic and Diastolic Native T_1_ Values*

Although the study population's mean MBF was lower in systole, this difference did not reach statistical significance because of the comparably large standard deviation of differences. This lack of phasic variation of MBF is in accordance with previous rest perfusion experiments.^38^, ^39^

Experiment 4 implies that a bias is introduced if native T_1_ values are not measured in the same cardiac phase as the perfusion series, which seems to be a direct consequence of the slightly different but strongly correlating systolic and diastolic native T_1_ values. However, since the corresponding bias and standard deviation of differences were rather small compared to the systolic‐to‐diastolic MBF variation and even more to MBF differences between segments with and without perfusion deficits, it seems reasonable to measure native T_1_ values in only one cardiac phase. Moreover, extrapolating from the mid‐ventricular short‐axis slice to the whole LV, the order of acquisition of slices in a clinical perfusion study, typically acquiring slices in different cardiac phases, should not play an essential role for MBF quantification.

Previous perfusion studies performing cardiac phase comparisons used the same systolic or diastolic AIF for MBF calculation to avoid potential AIF‐dependent effects.^38^, ^39^ The present study found no significant differences between MBF estimates determined using the same systolic AIF and MBF estimates determined using AIFs of the corresponding cardiac phases. Moreover, in contrast to MBF studies on healthy volunteers, the present study did not find that AIF_max_ was consistently lower in either systole or diastole.^38^, ^40^

### *MBF Quantification Using Regional Native Myocardial T_1_ Values*

Whereas there was no significant intersubject correlation between native myocardial T_1_ and global MBF values, MBF estimates tend to be lower if the native myocardial T_1_ values used for MBF estimation are higher (experiment 2). Consequently, MBF values of segments with perfusion deficits are overestimated and MBF values of segments without perfusion deficits are underestimated when using global instead of 6‐segmental native myocardial T_1_ values for nonlinearity correction. Moreover, while visually detected perfusion deficit segments demonstrated lower MBF values, irrespective of the employed native T_1_ values, the MBF difference between segments with and without perfusion deficits was largest in the case of MBF calculated with 6‐segmental native T_1_ values. It is therefore appropriate to measure native myocardial T_1_ values regionally in at least six segments to analyze possible perfusion deficits. Experiment 5 demonstrates that further T_1_‐sectioning of the myocardium leads to a reduced effect on pixelwise MBF estimates.

## Limitations

First of all, the patient number of the study was small and no healthy controls were investigated. Perfusion imaging was performed at rest only. However, since more than a third of the patients exhibited a rest perfusion deficit, a proof of principle was feasible without stress perfusion data. Findings on the influence of native T_1_ values on MBF estimates can be expected to also hold for stress perfusion experiments. The use of positron emission tomography (PET) perfusion imaging as a gold standard comparison was not feasible. However, the global MBF estimates obtained were in accordance with the results of other MR rest perfusion studies.

Only images at the same mid‐ventricular location were acquired, which was necessary for assessing the impact of systole and diastole on native T_1_ values and MBF estimates. However, it can be assumed that the main findings of the present study would apply to basal and apical short‐axis slices as well.

The MOLLI sequence is known to underestimate the T_1_ value; nevertheless, it was chosen for native T_1_ mapping due to its robustness and high signal‐to‐noise ratio.^26^ Furthermore, the presence of a small native T_1_ bias in all patients would not change the main findings of this study.

## Conclusion

Whereas cardiac phase‐specific native T_1_ values have a minor impact on MBF estimates, subject‐specific and myocardial segment‐specific native T_1_ values substantially affect MBF quantification.

## Supporting information


**Appendix S1** Supporting Information.
