# Supplementary material for: Impact of the Choice of Native T1 in Pixelwise Myocardial Blood Flow Quantification
Source: J Magn Reson Imaging. 2020 Oct 8;53(3):755–65. doi: 10.1002/jmri.27375 (PMC7891429; doi:10.1002/jmri.27375)
Supplement: Supplementary file 1 — Appendix S1 Supporting Information. [file JMRI-53-755-s001.pdf]

Supporting Information to The impact of the choice of native T_1 in pixel-wise myocardial blood flow quantification

Left ventricular function and late gadolinium enhancement imaging

For assessment of left ventricular (LV) function, retrospectively electrographically-(ECG)-gated, 2D-segmented balanced steady-state free precession (bSSFP) cine images were acquired in the LV two-chamber, three- and four-chamber views, as well as in contiguous short-axis slices covering the entire LV. Typical protocol parameters were repetition time (TR) = 3 ms; echo time (TE) = 1.3 ms; flip angle = 40°-45°; bandwidth = 1030 Hz/pixel; generalized auto-calibrating partially parallel acquisition (GRAPPA) factor = 2; field of view (FOV) = 270-315x360 mm^2; voxel size = 2.3×1.4×6.0 mm^3 for short-axis and 1.7×1.4×8.0 for long-axis series; and temporal resolution = 50 ms for short-axis and 36 ms for long-axis series, in both cases interpolated to 30 cardiac phases. Body surface area-normalized LV systolic function parameters were derived automatically by the scanner software, whereby the body surface area was calculated from patient's height and weight according to the Mosteller formula[1].

For assessment of late gadolinium enhancement (LGE) patterns across the whole LV, images in short- and long-axis orientations were acquired using a bSSFP-based inversion recovery sequence with phase-sensitive image reconstruction approximately 10 minutes after contrast agent application. The inversion time was optimized to null the signal intensity of remote myocardium. Typical parameters of the single-shot protocol employed in short-axis orientation were TR = 2.5 ms; TE = 1.1 ms; flip angle = 50°; bandwidth = 1184 Hz/pixel; GRAPPA factor = 2; FOV = 270-315x360 mm^2; voxel size = 2.6×1.9×8.0 mm^3. In long-axis orientation, a segmented protocol with TR = 2.9 ms; TE = 1.2 ms; flip angle = 50°; bandwidth = 977 Hz/pixel; GRAPPA factor = 2; FOV = 270-360x360 mm^2; voxel size = 2.0×1.4×6.0 mm^3 was used. The presence of subendocardial LGE in American Heart Association (AHA) segments not covered by the mid-ventricular short-axis slice was analyzed by three

[1] Mosteller RD. Simplified calculation of body-surface area. N Engl J Med. 1987;317:1098.

readers (CR, VN and UR with 4, 5 and 20 years of experience, respectively), where presence/absence of LGE was a majority decision.

Further details on the study population

Demographic details and systolic LV function parameters of the patients are given in Supporting Information Table S1.

LGE was identified in eleven of the 15 analyzed patients. In four patients, LGE was present, but not in AHA segments 7-12 of the mid-ventricular short-axis slice. Seven patients exhibited visual perfusion deficits, all of them being surrounded by LGE. No additional LGE segments not adjacent to visual perfusion deficits were found in the mid-ventricular short-axis slices.

Within the patients with perfusion deficits, three patients demonstrated a perfusion deficit in one segment, two patients demonstrated a perfusion deficit in two adjacent segments and two patients demonstrated a perfusion deficit in three adjacent segments.

Supporting Information Table S1: Demographic and systolic LV function parameters of the study population.

**parameter**	**mean ± SD**
age (years)	62 ± 7
height (cm)	173 ± 7
weight (kg)	83 ± 11
body surface area (m^2)	1.98 ± 0.16
heart rate (min^{-1})	61 ± 10
LV ejection fraction (%)	56 ± 12
LV end-diastolic volume index (ml/m^2)	87 ± 27
LV end-systolic volume index (ml/m^2)	40 ± 24
LV stroke volume index (ml/m^2)	47 ± 12
LV cardiac index ($L \cdot min^{-1} \cdot m^{-2}$)	2.8 ± 0.6

SD indicates the standard deviation.
